# The European Union’s governance of teachers and the evolution of a bridging issue field since the mid-2000s

**DOI:** 10.1177/14749041241234695

**Published:** 2024-03-05

**Authors:** Tore Bernt Sorensen, Xavier Dumay

**Affiliations:** School of Education, University of Glasgow, UK; GIRSEF, UC Louvain, Belgium

**Keywords:** European Union, governance, neo-institutional theory, field theory, teaching profession, teacher policy

## Abstract

Concerned with European Union (EU) governance of teachers since the mid-2000s, this paper makes an empirical as well as theoretical contribution to education policy studies in the context of EU governance. Drawing on neo-institutional field theory and an empirical material of policy documents and interviews, the paper analyses the consolidation and evolution of a field at the EU level that is focused on the governance of the teaching profession. We argue that this field constitutes a bridging issue field, spanning several policy domains, including education, employment and economy, and characterised by non-linear and relatively slow change. We demonstrate how the field since the mid-2000s has become elaborated via the strategic framing of teacher skills and careers as policy issues, the mobilisation of actors and networks, and an expanding institutional infrastructure of mechanisms and policy instruments. Theoretically, the paper advances the debate on EU governance by highlighting the epistemic gains of neo-institutional field theory in making sense of soft governance contexts and their trajectories as an outcome of the interplay between issue framings, different types of actors, and institutional infrastructure.

## Introduction

Teachers and teaching have since the 1990s become increasingly prominent on policy and research agendas globally, given momentum by the consolidation of a global education policy field (Lingard and Rawolle, 2011). This increasing interest in teachers is also reflected in European Union (EU) governance (Nóvoa, 2000; Sayer, 2006; Sultana, 1994; Symeonidis, 2021).

Drawing on neo-institutional field theory (Zietsma et al., 2017) and qualitative content analysis of policy documents and semi-structured interviews, this paper analyses the cross-sectoral developments in EU governance targeting teachers since the mid-2000s. We understand governance as relating to ‘a broad continuum of arrangements by which the behavior of groups and individuals is guided or coordinated’ (Assinger, 2020: 35). In the paper we use the terms ‘EU teacher governance’ and ‘EU teacher policy’ interchangeably to denote the range of EU arrangements and activities associated with teachers.

Our contribution to the literature is twofold. Empirically, the paper demonstrates that it is increasingly difficult to make sense of EU education policy on its own terms (Traianou and Jones, 2019) and that policy activities associated with teachers are pertinent in getting at the cross-sectoral implications of EU governance, since teachers as educators and a distinctive workforce are framed as important for the operation and performance of the EU as a political, economic and social entity. The paper argues that EU governance on teachers over the period from the mid-2000s to 2020 has come to form a ‘bridging issue field’ (Zietsma et al., 2017), blurrying the boundaries between education, employment and economic policy, and constituted by increasingly elaborated issue framings, a widening array of actors and an institutional infrastructure, which has evolved dramatically over the period. Furthermore, we argue that this distinctive bridging issue field is characterised by relatively slow and non-linear change.

Theoretically, the paper advances the debate on EU and global governance by highlighting the epistemic gains of neo-institutional field theory in making sense of soft governance contexts and their trajectories as an outcome of the interplay between issue framings, the mobilisation of different types of actors, as well as institutional infrastructure.

The paper proceeds with five sections. The next section introduces the theoretical framework and clarifies the research problem. Subsequently, a methodological section explains the operationalisation of theoretical concepts for empirical inquiry. Third, we present the findings regarding the scope of change in EU teacher governance, tracing the evolving issue framings, actor relations, and institutional infrastructures. The fourth section discusses our findings in terms of pace and linearity of field change. Finally, the conclusion reflects on the contributions of this paper and avenues for further research.

## Theoretical framework

In explaining the trajectory of EU teacher governance, our inquiry is guided by the question ‘what can one see by analysing this space as a field that one would otherwise not see?’ (Dubois, 2015: 209). The notion of ‘field’ has been used extensively in education policy studies, drawing on different theories which imbue the notion with different meanings. We draw on neo-institutional field theory, since it aligns with our interest in organisations as field actors and the evolution of different types of fields (Zietsma et al., 2017).

Important contributions to education policy studies have also drawn on the notion of ‘field’. While our paper in many ways builds on the insights from such previous studies, our use of theory and the concept of field reflect the specific objective of analysing the trajectory of policy production at the EU level, and how different policy domains are brought together to address issues associated with teachers and teaching. The entry point of our paper aligns with Robertson’s (2012, 2016; Robertson and Sorensen, 2018) argument concerning a shift from ‘thin’ to ‘thickening’ global governance of teachers since the 1990s and denationalising processes (Sassen, 2006) at work inside national territorial spaces and institutional domains. Developing Basil Bernstein’s theory for the analysis of global governance, Robertson points to the rescaling of governing technologies and the colonisation of the field of symbolic control by key agencies, such as the OECD, World Bank, and McKinsey and Company, ‘whose diagnosis and prognosis are part of a virtual circle between framing, representing, materializing, institutionalizing, and reproducing the modern teacher’(Robertson, 2016: 287). Focusing on the EU, our analysis concentrates on how the workings of this ‘virtual circle’ have hinged critically on the emergence and consolidation of a distinctive political field at the EU level associated with teachers and teaching, and the nature and constituents of this field.

Another strand of education policy studies has adapted Pierre Bourdieu’s theory of social fields to a more globalised governance environment (Lingard et al., 2005; Lingard and Rawolle, 2004, 2011; Mangez and Hilgers, 2012). Whereas Bourdieu’s field theory has the capacity to explain the evolving relations between economic and cultural capital in the field of power, the perpetual struggle and competition in different social fields, the relative authonomy of fields vis-à-vis other fields, and the features and actions of individuals with reference to different types of capital, neo-institutional theory is more sensitive to the role of organisations and how they relate to each other (Martin, 2003; Zietsma et al., 2017). Still, this paper aligns with the arguments of Bourdieusian studies concerning the emergence of a global education policy field subject to demands for global economic competitiveness and the rescaling of authority beyond national spaces. The paper also hints at the increasing influence of private sector enterprises and public-private partnerships in policy formation. However, rather than understanding education as a separate policy domain, the relative autonomy of which has become reduced due to ‘cross-field effects’ from the fields of the economy and journalism (Lingard and Rawolle, 2004; Lingard et al. 2005), this paper broadens the scope and situates the governing of teachers as part of public policy (Dubois, 2015). This is an important premise for our analysis, as economic arguments, for instance, have underpinned EU policies on teachers since the 1990s (Husén et al., 1992; Neave, 1992; Nóvoa, 2000; Sultana, 1994). As the paper shows, the more important development in the context of the EU concerns how different logics have been brought together in the framing of teacher policy issues, along with the mobilisation of different types of actors, and an expanding institutional infrastructure.

Another important difference between the studies drawing on Bernstein’s and Bourdieu’s theories and this paper concerns their differing emphases on power, struggle and conflict. We do certainly not dispute the presence and importance of power and interest advocacy in political fields. However, the neo-institutional concept of structuration (DiMaggio and Powell, 1983; Martin, 2003) at the same time refers to the socialisation and alignment between different types of organisations operating in a specific field, which might be the result of increased interaction over longer periods of time and the development of an awareness among field actors that they are involved in a common enterprise, implying a sense of shared identity. In neo-institutional theory, fields evolve via decision-making processes among organisations that, albeit having different objectives, find it necessary and advantageous to interact with one another (Wooten and Hoffman, 2008). Fields are hence understood as socially and relationally constituted; they are made up of actors and their relations, structured to some degree around common meanings and interests, including a working consensus regarding the ‘nature of the game’ that actors are involved in (DiMaggio and Powell, 1983; Martin, 2003). We would argue that this conception of fields and the interests involved is critical for understanding the mobilisation of different types of organisations in EU teacher governance over recent decades.

The ambition to further a transversal and comprehensive understanding of teacher governance as public policy, and hence to avoid the tendency to understand policy domains as ‘silos’ (Peters, 2015: 1), aligns with recent scholarship on boundary-spanning policy regimes (Graf et al., 2023). Yet, neo-institutional theory offers a set of concepts and hypotheses which allow for theory-building via the analysis of different types of fields and their trajectories of change.

In their comprehensive review, Zietsma et al. (2017) observe that the recognition of differences between field types is central for theorising field agency and evolution. Specifically, the formation of bridging issue fields implies a policy-led drive to address issues that involve several policy domains. Bringing together a diverse set of actors with distinct identities and commitments, the central activity in bridging issue fields concerns the negotiation and contestation of how policy issues are framed, and which actors are to have access to the field. Multiple and conflicting logics thus tend to be present in bridging issue fields (Hoffman, 1999; Zietsma et al., 2017).

Moreover, bridging issue fields constitute a policy-led mechanism for adjustment and innovation in professional exchange fields. This type of field concerns a focal profession and its interaction with exchange partners, such as the organisations in which professionals work, other professions they engage with, regulators, et cetera (Zietsma et al., 2017). Building on this idea, teachers, for instance, feature in a distinctive professional exchange field, the purpose of which is to control professional practices and boundaries around teaching. Such a field would typically include, in addition to teachers as the main profession, trade unions, professional associations, school leaders, teacher educators, employers, government authorities and other actors. The notion of a professional exchange field for teachers implies that we do not understand EU teacher governance as simply nested within a wider field of education governance. While EU education governance involves a strong ideational component of the knowledge-based economy and employability (Antunes, 2016; Lawn and Grek, 2016; Robertson, 2008; Walkenhorst, 2008), suggesting the relevance of analysing it as a bridging issue field, this paper’s focus on EU’s governance of teachers means that the analysis concerns a particular bridging issue field, constituted by a specific set of actors and issue framings, and combining education, economic as well as employment policy in a distinctive way.

In addition, Zietsma et al. (2017) argue that institutional infrastructure helps to determine field evolution, yet they do not go into much detail on this point. Given the importance of organisational or institutional elements in shaping interaction around policy issues, we wish to highlight such institutional infrastructure in our theoretical framework (Borrás and Radaelli, 2011). Institutions, in the sense of policy mechanisms, procedures and instruments, serve to garner attention towards certain issues, potentially across different policy domains, and they form integrative forces in a given political field when invested with authority by actors (Jochim and May, 2010).

Considering existing evidence, our hypothesis is that EU teacher governance since the mid-2000s has come to form a bridging issue field. Evidently, teachers are as educators key actors in education sectors as well as workers operating in labour markets. Like other professions, the teaching profession is directly affected by policies of the sector in which they work as well as employment and labour market policies. Yet, this is a separate point from whether EU teacher governance has involved the creation of a bridging issue field which explicitly bring together the silos of different policy domains. The period from the mid-2000s onwards is particularly interesting, since the issue salience of teachers and teaching in EU governance gathered momentum at this point (Caena, 2014; Stéger, 2014; Symeonidis, 2021). Moreover, an EU schools policy was launched in 2007, and teacher governance has increasingly become subject to cross-sectoral coordination via, for instance, the European Semester (hereafter ‘the Semester’) (Sorensen et al., 2021; Symeonidis, 2021). Launched in 2011 as part of Europe 2020, the Semester is the EU’s annual cycle for socio-economic governance. The Strategic Framework for European Cooperation in Education and Training (ET2020) was from 2012 onwards incorporated into Europe 2020 by including education policy issues in the Semester (Eeva, 2021). Finally, we note that the Commission tends to set the agenda in EU teacher governance via its Communications (Sorensen et al., 2021; Stéger, 2014; Symeonidis, 2021), underpinned by its increasing capacities in collecting, producing and diffusing policy knowledge in the form of statistics, indicators and comparative research (Grek, 2016; Lawn and Grek, 2012), and the mobilisation of actors and creation of expert networks and working groups (Normand, 2010; Stéger, 2014).

## Research design

The paper is guided by a set of research questions concerning the scope, pace and linearity of change in the field of EU teacher governance at the European level since the mid-2000s (see Table 1). Our empirical inquiry involves the analysis of sixteen policy documents and nineteen interviews (see overview in Appendix). The document analysis was particularly important for identifying timelines of events, policy developments and references to actors, whereas the interviews clarified processes and relations between issues, institutional infrastructures and actors.

**Table 1.** Research questions.

Field-level changes in EU teacher governance
*Scope of change*
➢ How have the field constituents of EU teacher governance, that is, issue framing, actors, and institutional infrastructures, changed between 2007 and 2020?
*Pace of change*
➢ At which speed has the field of EU teacher governance changed over the period?
➢ Has the field developed incrementally or in revolutionary ways?
➢ Have some field elements changed faster than others, and what have triggered these changes?
*Linearity of change*
➢ Do the field elements in EU teacher governance move in the same direction at the same pace?
➢ To which extent has the field been characterised by contestation, competition and reversals of directions?

The selected policy documents consist of Commission Communications and Staff Working Documents recapitulating EU activities and the Commission’s preferences in a relatively detailed manner. Issued in the period 2007–2020, the selected documents relate to the main EU agendas of the Lisbon Strategy and Europe 2020 and the strategic frameworks for European cooperation in education and training (ET 2010 and ET 2020). The documents were selected on the basis of existing research (Antunes, 2016; Sorensen et al., 2021; Stéger, 2014), extensive mapping of documents, and recommendations from Commission officials concerning key documents. Staff from DG Education, Youth, Sport and Culture (DG EAC) singled out especially the Communication *Rethinking Education: Investing in skills for better socio-economic outcomes* (EC, 2012a) and the associated Staff Working Document *Supporting the Teaching Professions for Better Learning Outcomes* (EC, 2012b), as well as the Communication *School development and excellent teaching for a great start in life* (EC, 2017a) along with the related Staff Working Paper (EC, 2017b).^1^ In addition, we have included documents covering the entire period in order to trace developments in a more fine-grained manner. The primary criterion for selection among the numerous other Communications and Staff Working Documents issued by the Commission over the period was that a document includes contents explicitly addressing teachers and teaching. Such contents range from single paragraphs and sections to entire documents.

In addition, nineteen realist theory-laden semi-structured interviews (Pawson, 1996) were conducted with individuals with current or recent first-hand experience of EU policymaking. The participants were primarily selected via purposive sampling, considering their positions in different organisations taking part in EU level governance. The study also benefitted from snowballing, where participants suggested other potential interviewees (Tansey, 2007).

Considering the research questions and the nature of the empirical material, we developed an analytical strategy with two stages. First, we coded the empirical material with a focus on the three field constituents of issues, actors and institutional infrastructure. Second, we developed second-order coding and interpretations, discussing what our findings mean in terms of the pace and linearity of change. For these purposes, the study adopted qualitative content analysis (Mayring, 2014). Distinguished by systematic procedures of text analysis, qualitative content analysis involves interpretative yet rule-based assignment of categories to the empirical material. Given direction by the research questions, our content analysis concentrated on the specific system of categories which encapsulate central aspects in the empirical material, combining the techniques of inductive category formation and deductive category application in a series of iterative steps. Accordingly, the deductive category application included in the coding agenda (see Table 2) was created after a prolonged period of desk research, literature review and analyses of the empirical material involving inductive category formation, identification of themes and actors and lists of keyword frequencies and cross-references. In this respect, the deductive analysis involved the steps of first defining the category system, pilot analysis, revision of categories and rules of application, followed by analysis of the full material, including continuous interpretation of contingencies. The two authors of this paper tested reliability in applying the coding agenda, following the standard procedure for checking inter-coder agreement, both of us analysing a sample of two documents and two interviews (i.e., 11–13% of empirical material) (Mayring, 2014).

**Table 2.** Coding agenda for deductive qualitative content analysis.

Analytical category	Guiding questions	Variables
1. *Issue framings* The definition of problems, objectives and solutions (Furnari, 2018; Jochim and May, 2010)	‘How are teacher policy issues framed?’ ‘Has the framing of teacher policy issues changed over the period?’	a. Diagnostic frames: Which teacher policy problems are represented? b. Prognostic frames: Which solutions are suggested to ameliorate the policy issues? c. Levels of issue framing: education in general or teachers specifically (coding one category only) d. Logics (possible to code more options) • *Teacher education and lifelong learning logic* (teacher education, professional development, competences, etc.) • *Employment logic* (attractiveness of profession, teacher skills, labour markets, careers, teacher recruitment, working conditions) • *Economic logic* (funding, salary and financial incentives) • *Governance logic* (regulation, evaluation and accountability of profession)
2. *Actors and relations* The unfolding relations between different types of field actors, drawing on Zietsma et al.s’ (2017) inventory.	‘Which actors are mentioned in interviews and documents, how are their relations and roles in EU governance represented, and how do these change over the period?’	a. Type of actors (possible to code more options) • *Boundary organisations* with central roles in governing the field, managing formal and informal field boundaries vis-à-vis other fields, and diffusing ideas and innovations • *Formal units of EU governance*, e.g. member state governments and the Commission • *Arbiters of taste*: organisations reputed for their expertise, rankings, and awards • *Field coordinators*: professional associations, teacher unions and employers • *Institutional entrepreneurs* • *Civil society organisations* • *Competing systems* b. Status of actors: central/peripheral/middle status (one category only)
3. *Institutional infrastructures* Institutions contribute to the strength and governing capacity in a given political regime, or field, by structuring and channelling authority, attention, resources and information flows (Jochim and May, 2010) Our framework draws on Milana and Klatt (2020) and Marques (2021)	‘Which elements of institutional infrastructures in EU teacher governance are mentioned in the interviews and documents, and have they changed over the period?’	a. Types of institutional infrastructure (coding one category only) • *Fundamental EU institutional arrangements*, e.g. EU Treaty and distribution of competences • *Policy mixes*, including strategies and policies • *Governance mechanisms* involving sets of formalised procedures, processes and coordination • *Policy instruments* b. Types of policy instruments (coding one category only) • *Coordinated working groups/networks* • *Peer learning arrangements* • *Data generation* • *Benchmarks* • *Funding schemes and incentives* • *Other instruments* c. Policy domain of institutional infrastructure (possible to code more options)

## Analysis and findings

This section presents our findings concerning the scope of change in EU teacher governance. Three complementary sub-sections highlight the evolution of issue framings, actors and institutional infrastructures since the mid-2000s, respectively.

### Issue framings

Overall, teacher policy has helped to substantiate the Lisbon and Europe 2020 strategies. The launch of a EU school policy (EC, 2007a, 2008a, 2008b) and teacher education agenda (EC, 2007b) signals the reinforced political focus on teaching and the teacher workforce in the latter years of the Lisbon Strategy. To realise the objectives of building a knowledge based economy, the Lisbon Strategy was relaunched in 2005, furthering the emphasis on jobs and growth and calling for mobilisation of all levels of education and training. Already at this point, the Commission’s discourses highlighted the educational and economic implications of teacher policy, with teachers being framed as ‘key agents for a change’ not only as educators but also as an important workforce for the EU’s economic competitiveness (EC, 2007a: 9).

**Figure 1.**
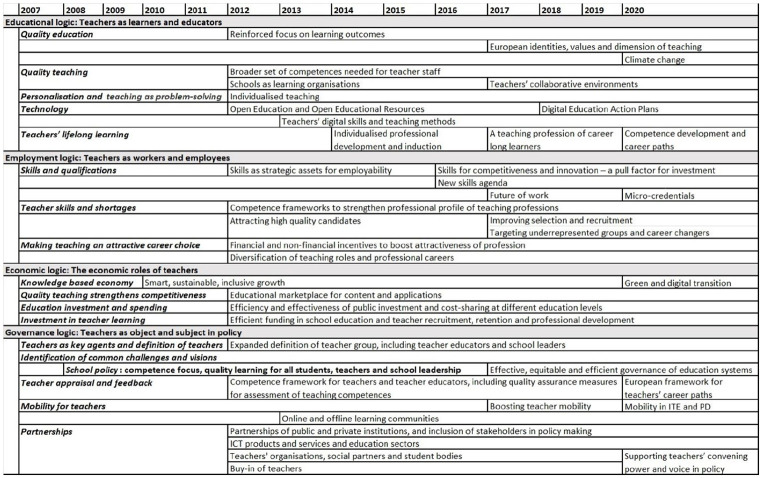
Logics and issue framings of EU teacher policy.

Our findings demonstrate the continuity and elaboration of policy issues related to teachers in EU governance. In line with the coding agenda (see Table 2), Figure 1 provides an overview of the main issue framings of EU teacher policy, distinguishing between education, employment, economic and governance logics. The Figure indicates that the main issue framings were introduced, or already evident, with the set of Commission policy documents published in 2007 and 2008. Subsequently, the *Rethinking Education* Communication (EC, 2012a) and the associated Staff Working Document on the teaching profession (EC, 2012b) were decisive in adding new dimensions to these issue framings. This elaboration continued over the period until 2020, in the process furthering the mutual implications between issue framings already in place and effectively consolidating teacher policy as a bridging issue.

#### Governance logic

Concerning the regulation of the profession, the issue framings included in Figure 1 might be summarised in three analytical points. First, as a matter of definition, the group of teachers were initially defined as those with the status of teachers in general education and vocational education, according to legislation and practice of a Member State, excluding persons employed outside formal education and training systems (EC 2007a, 2007b). However, the importance of teacher educators was acknowledged from the outset (EC, 2007b). This was subsequently reflected in the broader definition: ‘The teaching professions include all those who teach, educate, manage learning, or educate educators in the following education fields: early childhood education and care; compulsory education; vocational education and training; and adult education’ (EC, 2012b: 5).

Second, the teaching profession is framed as both object and subject of reform; while reforming the teaching profession serves as means in pursuing EU objectives, the profession is also to be consulted as part of policy-making. This relates to the issue of partnerships, elaborated during the 2010s for the mobilisation of funding, mutual learning and joint policy development and implementation. Such partnerships are to involve all public and private stakeholders, including teachers’ organisations, social partners as well as teachers in general (EC, 2012a; A9, A11).^2^

Third, the Commission frames the challenges as well as the solutions regarding the teaching professions and school systems as similar across Europe: ‘The challenges facing the teaching profession are, in essence, common across the European Union. It is possible to arrive at a shared analysis of the issues and a shared vision of the kinds of skills that teachers require’ (EC, 2007b: 12). In terms of school policy, more European cooperation was deemed necessary, ‘given the common nature of many of the challenges facing school systems and the importance of these issues for the Union’s socio-economic future’ (EC, 2008: 4). By 2017, EU school policy had evolved to directly address the governance of school systems, represented as involving similar – again – challenges across Europe, including regarding the effectiveness and efficiency of expenditure (EC, 2017a, 2017b). Due to the principle of subsidiarity, the Commission needs to be careful in the representation of common European issues and provide services that are ‘cross-border’ in nature, that is, activities which do not duplicate those of member states but add new perspectives and capacities to their existing arrangements (A9, A11). At the same time, the very diversity of conditions in member states calls for tailoring analysis and policy recommendations sensitive to national circumstances. Accordingly, EU teacher policy during the 2010s has involved increasingly specific application of issue framings to member states, indicated by terms such as ‘tailor-made’ and ‘demand driven technical support’ (EC, 2012b: 58, 2017a: 10). This evolution is encapsulated by the Semester, where teacher related issues have been mentioned for many member states (A11).

#### Education and employment logics

Our findings suggest that the issues of quality teaching, attractiveness of the teaching profession, and teachers’ skills and careers have become mutually implicated, together constituting an evolving cluster of bridging issues, which mix education and employment logics. This cluster of issues have become elaborated since the late 2000s.

First, concerning quality teaching, teacher competences and qualifications were framed as ‘vital to the achievement of Lisbon goals’ since ‘teacher quality is the most important within-school factor affecting student performance’ (EC, 2008a: 11) and ‘the quality of teaching is one key factor in determining whether the European Union can increase its competitiveness in the globalised world’ (EC, 2007b: 3). In line with the educational logic, the Commission advocated policies to improve the quality of teacher education, lifelong learning, skills and qualifications, as well as calling for teachers to take charge of their learning pathways and engage with research (EC, 2007b).

Second, the attractiveness of the teaching profession is a longstanding policy issue in most member states, with specific challenges shaped by salary levels, ageing of the workforce, and its status in society and in contractual terms, including whether teachers enjoy civil servant status (EC, 2007b; A1, A7). The efforts to make the teaching profession more attractive involve, again, a mixing of education and employment logics, including competences and skills, professional autonomy, lifelong learning, mobility, career progression, working conditions, wages, job satisfaction and entrepreneurship and innovation in schools and teacher education (A6, A7, A11, B1). These issues manifest themselves differently depending on the educational level, from early childhood to higher education (A3).

Third, incentives and investment in teacher education and professional development were initially represented – consistent with the educational logic – as the main lever for improving the competences, professionalisation and attractiveness of the profession (EC, 2007b, 2008a). However, during the 2010s the concept of skills became the key term in Commission discourses to frame competences and qualifications in terms of preparation for employment and labour markets, for teachers as well as for students, with the attractiveness of the profession being represented as instrumental in addressing teacher skills shortages across Europe (A1, A5, A7). Marking a pivotal moment in the framing of skills and employment preparation, the Communication ‘Rethinking Education’ points out that in the context of ‘sluggish economic growth’, ‘fast-rising youth unemployment’ and ‘a shrinking workforce due to demographic ageing’, the most pressing challenges for member states are to address the needs of the economy and ‘delivering the right skills for employment’ (EC, 2012a: 2).

Finally, the issue of teachers’ careers has become elaborated over the period, recently leading to a European framework (EC, 2017a, 2020b; see European Commission, 2020). Initially mentioned in purely descriptive terms, teachers’ careers have since 2012 become subject to more nuanced discussion of specific stages, promotion and advancement, diversification of pathways as well as horizontal differentiation, including new roles requiring different sets and levels of competences, responsibilities and salary. In other words, teachers’ careers have come to mix logics of education (lifelong learning, competences and quality teaching), employment (career stages, remuneration, recruitment, the widening range of job tasks and responsibilities), and governance (assessment, competence frameworks and quality assurance) (EC, 2012a, 2012b, 2017b).

Yet, the career issue also indicates boundaries associated with the EU’s political mandate. Teacher salary levels, for instance, remain a too sensitive issue for member states to be addressed directly with EU level actions (A9). Still, drawing on the increasing production of comparative data, salary levels, differences and cuts have become discussed in more detail (EC, 2007b, 2012a, 2012b, 2017b), including in the 2019 edition of the ‘Education and Training Monitor’, which focused especially on teachers (European Commission, 2019). Moreover, while contractual issues are little discussed, the framing of careers suggests the marginalisation of the notion of civil servants, last mentioned in 2012 (EC, 2012b).

#### Economic logic

The economic arguments related to EU’s governance of teachers were also elaborated during the 2010s. These arguments work both ways, including why teachers are important for the economy, as well as why sustained investment and spending are imperative for quality learning and teaching.

Quality teaching has thus throughout the period been represented as growth-enhancing and important for the EU’s economic competitiveness, with skills being framed as a pull factor for investment (EC, 2012a, 2016b, 2017a). In the early 2010s, the commercial opportunities of an educational marketplace for content and applications emerge as a new issue, in line with the promotion of entrepreneurship education, partnerships between education, business and research, and ICT based communities of practice for teachers (EC, 2012a, 2013). The Digital Education Action Plans of 2018 and 2020 have since further advanced these issue framings (EC, 2018a, 2020a).

Vice versa, the issue of investment needs in education was elaborated during the 2010s, focusing on the double challenge of prioritising public spending in education and finding smart ways of deploying financial resources (EC, 2012a, 2020b). Whereas policy documents issued in 2007-2008 include generic calls for sustained investment in education sectors, subsequent documents, in the context of the financial crisis putting pressures on budgets, reinforced the focus on the effectiveness and efficiency of public investment as well as public and private cost-sharing. Given the large proportion of school budgets spent on teachers, effective teacher recruitment, retention and professional development were singled out as means for important returns of investment (EC, 2012a, 2012b).

### Actors

This subsection demonstrates the mobilisation in EU governance since the mid-2000s of a diverse and widening range of actors with an interest in teachers. Not intended to be exhaustive, the account concentrates on the most central types of actors: formal units of EU governance (including the Commission as the main boundary organisation), arbiters of taste, institutional entrepreneurs, and field coordinators (cf. Table 2).

#### Formal units of EU governance

Our analysis suggests that the Commission, member state governments, and the Council formations of the EU,^3^ are the most central actors in the field, and in this order. Meanwhile, the European Parliament remains peripheral in its institutionalised role of consultation at a stage in the policy process, when initiatives have already been framed. The European Economic and Social Committee (EESC) represents another formal unit of EU governance that is peripheral though it has interests in the field.

The pursuit of policy-led change regarding teachers depends on the commitment of member state governments. Member state governments and administrations remain central in the field, due to their political mandate as well as providers of case policies and data (A1, A5, A7, A8). Meanwhile, the central role of the Council formations of the EU is evident in the number of references to Council Conclusions in Commission documents about teachers. Moreover, the European Council issues the CSRs in the Semester, and several member states have used their Council of the EU Presidencies to advance teacher policy issues (A8, A11). Interestingly, the analysis indicates an evolution in the engagement of Ministers of Finance and Education in EU teacher governance. Following the financial and economic crisis of the late 2000s, Ministers of Finance were more central in shaping EU debates on education and teachers than Ministers of Education, due to strong focus on public budgets during this period (A2, B1). However, Ministers of Education and the Education Council of the EU have become more visible since the mid-2010s, highlighted by the Joint Council meeting with Education and Finance ministers in November 2019 on investment in education, and the fact that numerous Ministers of Education have taken part in the annual European Education Summits launched in 2018 (A8, A11).

Our analysis corroborates existing studies concerning the Commission as the most influential actor in the EU’s governance of teachers. While the Commission must consider the limited ‘support competence’ of the EU in education (Walkenhorst, 2008) and relations to member state governments and the Council formations of the EU, the Commission has the right to spend its own budget and continuously explores avenues for increasing its influence in EU education and teacher governance via activities with a strong employment and skills dimension, such as the European Pillar of Social Rights, the Semester and social dialogue (A1, B1, C1). In this respect, an interest organisation representative pointed to the importance of the Commission issuing the ‘draft zero’ documents that subsequently come to drive policy, funding and lobbying (C1).

Specifically, we find that the Commission is the major boundary organisation in the bridging issue field of EU teacher governance, seeking to maintain and redefine field boundaries towards other fields, including the professional exchange fields of teachers, via the framing of issues, inclusion and exclusion of actors in policy processes, and orchestration of institutional infrastructures. The following paragraphs show that the Commission’s role as boundary organisation is reflected both in its internal organisation and the mobilisation of a widening and diverse array of actors with an interest in teachers.

Concerning internal relations, during the 2010s the cross-sectoral coordination between Commission Directorate-Generals (DG) was improved for the purpose of building country-specific expertise, monitor policy issues and reforms, and tailor recommendations to member states (A1, A5, A7, A9). DG Education, Youth, Sport and Culture (DG EAC) has the lead on teacher policy issues, yet several DGs have become interested in monitoring issues related to the teacher workforce, such as DG Employment, Social Affairs and Inclusion (EMPL) on employment conditions and DG Economic and Financial Affairs (ECFIN) concerning education budgets and teacher salaries, altogether resulting in more cross-sectoral coordination and communication between DGs (A7).

These coordination efforts are evident in the Semester. Involving virtually all DGs, this major mechanism of socio-economic governance is led by the Secretariat-General, DG ECFIN, DG EMPL, and DG Internal Market, Industry, Entrepreneurship and SMEs (GROW). For DG EAC, DG EMPL is the main hub for coordination, with the latter having a larger capacity for country-specific analysis and a stronger institutional mandate for socio-economic governance (A4, A5). In the interviews, the strong representation of education-related issues in Semester Country Reports and Country-Specific Recommendations (CSR) (in 2019, the CSRs included educational issues for all member states) was understood as an indication of DG EAC’s relative success in highlighting issues. DG EAC staff is also involved in drafting and ‘consistency checks’ of Semester materials, such as the Country Reports issued by the Commission to member states, as well as the cross-DG ‘Country Teams’. Consolidated during the mid-2010s, these Country Teams encapsulate the Commission’s role as boundary organisation, as they via monthly meetings would develop the country-specific expertise required by the Semester mechanism and liaise with member state governments and stakeholders, including also the ‘Fact-Finding Missions’ to member states that form part of the Semester cycle (A5, A7, A8, A9).

The Country Teams and Fact-Finding Missions reflect the Juncker Commission’s priority from 2015 onwards to renew social dialogue and build partnerships with a widening array of actors. The Commission has increasingly consulted, formally and informally, member state administrations, field coordinators and civil society organisations both at EU and member state levels. In this way, the Semester has been ‘opened up’, from the initial focus in the early 2010s on relations with Ministers of State and Finance, to later also include consultations with Ministers and Departments of Education (A2, A4, A5, A6, B1, C1). Field coordinators, such as European Trade Union Committee for Education (ETUCE), have facilitated such consultation by sharing details of member state-based affiliates with the Commission (A9, B1, C1). Indicating its increasing capacity to consider the specificity of sites, the Commission has begun to reach out to regional authorities in the Semester. This is an important finding calling for further studies, given the devolution of powers in several member states, such as Germany, Portugal and Spain, in matters of education and teacher policy.

In terms of external relations, the Commission has drawn on and sought to engage a diverse array of actors in EU policy-making. Below, these are categorised as arbiters of taste, institutional entrepreneurs and field coordinators.

#### Arbiters of taste

EU teacher governance has over the period drawn on different arbiters of taste. These relations change over time. Virtually absent in recent policy documents, the consultancy McKinsey & Co. was a prominent arbiter of taste in the early stages of EU teacher policy, indicated by substantial references in the first published Commission documents in the empirical material (EC, 2007b, 2008a, 2008b; A1). At the same time, the analysis confirms existing evidence about the Organisation for Economic Co-operation and Development (OECD) as a continuous reference point in EU governance in terms of policy-driven research, indicators and statistics on teachers and education more generally (Grek, 2016; Lawn and Grek, 2012; Sorensen and Robertson, 2020). The interviews point to the unrivalled cachet of the OECD globally in these areas and the deepening collaboration between the Commission and the OECD, although a handful of EU member states are still not members of the OECD (A1, A2, B2, C1). Moreover, references to OECD projects and its Teaching and Learning International Survey (TALIS) and Programme for International Student Assessment (PISA) are ubiquitous in the analysed body of Commission documents. At the same time, our analysis suggests important developments. Whereas their collaboration in the 2000s primarily involved the Commission providing vital funding for OECD programmes such as TALIS, in return bolstering the status of the Commission on matters of teacher policy, the Commission has since developed its own capacity for epistemic governance regarding education and teachers (A1). The clearest example is the *Education and Training Monitor* series issued annually by the Commission since 2012. The 2019 edition (European Commission, 2019) had teachers as the key theme, including horizontal EU analysis as well as country reports for each member state. The publication was launched at the 2019 European Education Summit, which also focused on teachers, a decision reflecting teachers’ central role for the European Education Area and the opportunity to draw on new data from the OECD TALIS 2018 survey (A11). In this sense, the Commission has also taken on the role of arbiter of taste, further indicated by initiatives such as e-Twinning Ambassadors, European Innovative Teaching Award (EC, 2020b), and the ambition of ‘modelling’ best practices in governance and stakeholder inclusion for member states (A11).

#### Institutional entrepreneurs

The global network organisation Teach For All stands out as an organisation that via lobbying in Brussels and collaboration with high ranking profiles, such as Xavier Prats Monné, former DG EAC Director General, and Andreas Schleicher, OECD Director for Education and Skills, has managed to direct attention towards its approach to teacher education, leadership and careers. This approach is singled out by the Commission as innovative due to its strong focus on transversal skills and for allowing different careers over a lifetime. During the 2010s, Teach For All has expanded across numerous member states, and the increased visibility has resulted in regular contacts with Commission staff and ET 2020 projects on teachers (A1, A6, A11).

Moreover, the repeated calls in the analysed policy documents for digital innovation and edu-technology from 2012 onwards align with the EU’s promotion of public-private partnerships and entrepreneurship (Leffler 2009; Souto-Otero 2019). In the empirical material, these evolving educational marketplaces constitute the only area where external competition is highlighted, pointing to the need for supporting entrepreneurs in Europe to better compete with industries based in the US and China (EC, 2013, 2017b). Specifically, interview participants mentioned European Schoolnet, a network of 32 European Ministries of Education, as an important actor due to its role in DG EAC and DG REFORM activities (A12), as well as the deep interest in teachers among multinational enterprises such as Google and Microsoft (B2). The full range of institutional entrepreneurs is much wider, indicated by the creation of networks such as the ‘Grand Coalition for Digital Jobs’, which included, for instance, the European Federation of Education Employers (EFEE) and European Schoolnet, and where teachers’ digital skills were among the issues to be addressed (EC, 2013). The initiative was expanded in 2016 with the ‘Digital Skills and Jobs Coalition’, including more than 400 organisations from the public, private and non-profit sectors (EC, 2018a).

#### Field coordinators

Some of the field coordinators might also be understood as entrepreneurs. Including professional associations, teacher unions and employers, the mobilisation of field coordinators are crucial for connecting EU policy with practice in member states. We find that field coordinators have become more visible in EU teacher governance since the mid-2000s, though their influence remains limited. Characteristically, several field coordinators, such as EFEE, Association of Teacher Education in Europe (ATEE) and European Network on Teacher Education Policies (ENTEP), the latter formally launched in 2000 and composed by a mixture of ministerial civil servants and higher education experts, have been created as a result of EU activities and funding programmes (Poissonneau and Nolda, 2012; Sayer, 2006), with some of them later becoming members of ET 2010 and ET 2020 Working Groups (A1, A11, E1).

As counterparts in the European Sectoral Social Dialogue in Education (ESSDE), EFEE and European Trade Union Committee for Education (ETUCE) stand out among the field coordinators. The launch of the ESSDE in 2010 required that ETUCE and EFEE were granted the formal status of European social partners in education by the Commission, providing them consultation rights in EU policymaking not bestowed on other interest organisations. The Commission (EC, 2017a, 2017b) singles out the ESSDE as a pertinent policy forum in the context of its initiative to reinforce peer learning with a focus on teachers and school leaders’ careers and professional development. Moreover, EFEE and ETUCE have been members of several ET 2020 Working Groups, including the one on schools and teacher policy, and they take part in EU cross-sectoral social dialogue, which involves ETUC, CEEP and BusinessEurope as the main social partners (B1, B2, C1; see also Sorensen and Dumay, 2023).

Importantly, access for professionals to the field does not necessarily have to take place via the representation of field coordinators. Reflecting its aspirations to foster ‘buy-in’ from the teaching profession and model inclusive forms of governance, the Commission has during the 2010s increasingly reached out to teachers directly, for instance via digital platforms, the European Education Summits (around 150 teachers took part in the 2019 Summit), the European Innovative Teaching Award and e-Twinning Ambassadors (EC, 2020b; A9, A11).

In summary, the findings indicate an expansion and strengthening of the actor relations in the EU’s governance of teachers since the mid-2000s. In particular, the analysis highlights the mutual implications between the Commission’s internal and external relations, and that the development of cross-sectoral coordination in the Commission together with its mobilisation of actors and networks have effectively changed the constitution of the bridging issue field since the mid-2000s. The evolution of this field has resulted in stronger linkages between EU policy and practice, or in other words, an increased capacity of the bridging issue field to generate adjustment and innovation in the professional exchange fields of teachers.

### Institutional infrastructures

**Figure 2.**
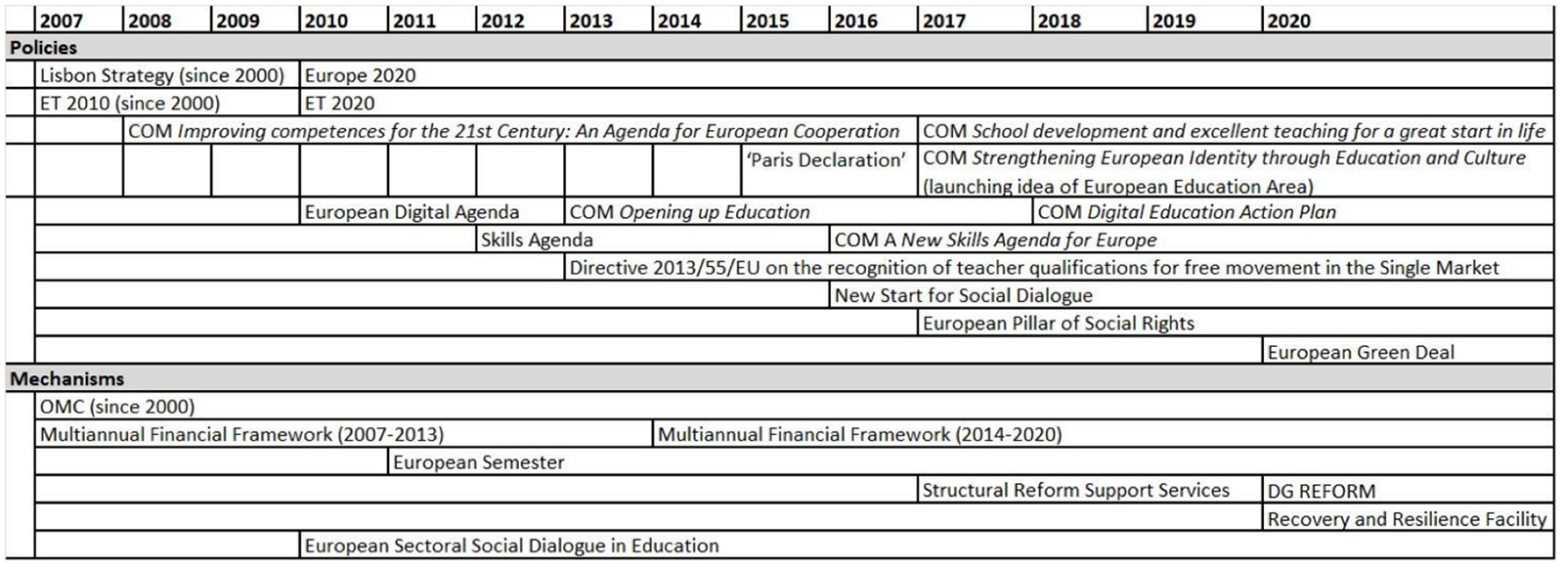
Policies and mechanisms in the field of EU teacher governance.

**Figure 3.**
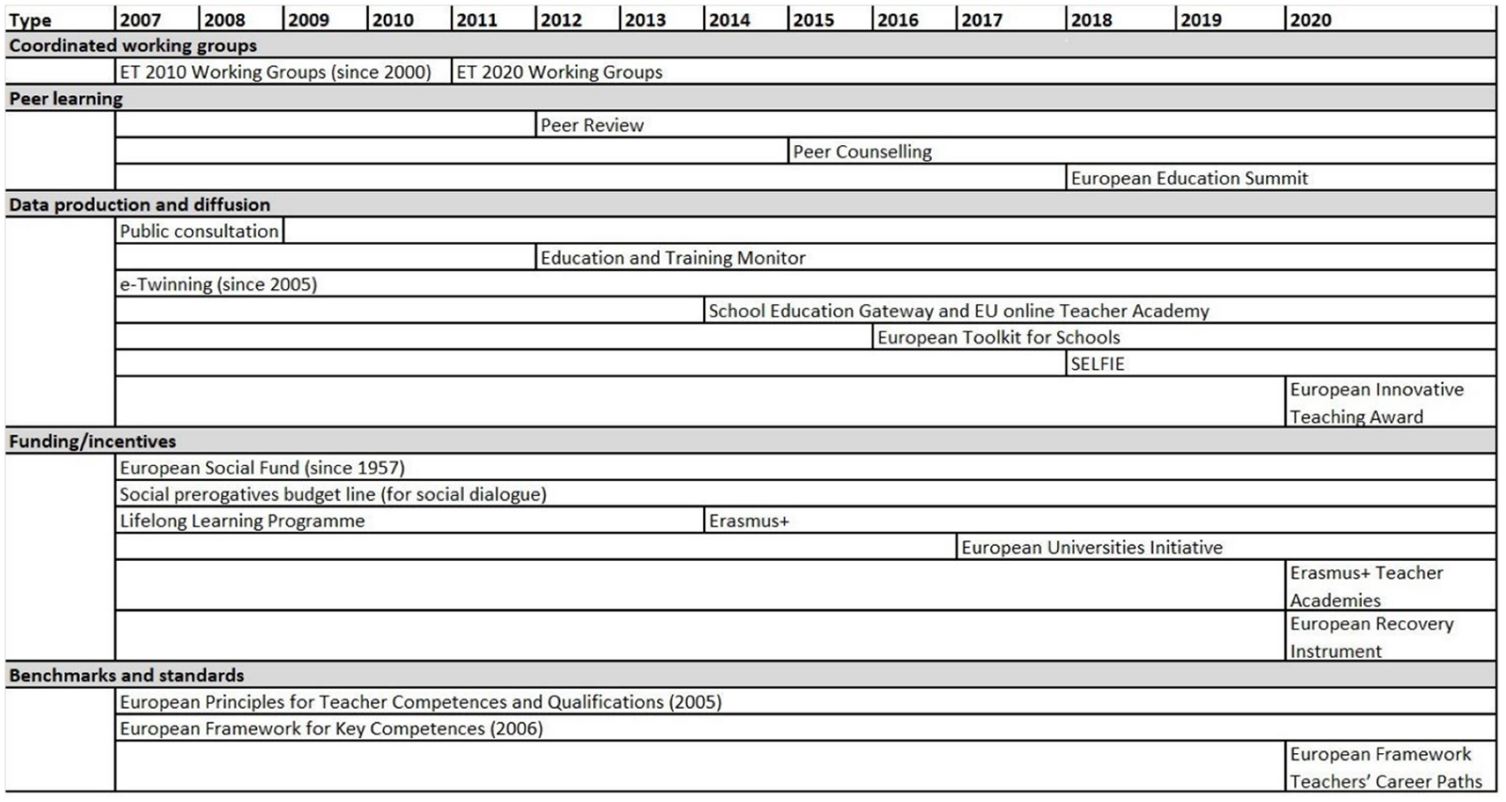
Instruments in the field of EU teacher governance.

The findings demonstrate an elaboration of institutional infrastructure in EU teacher governance since the mid-2000s, suggesting an increasingly complex and structured institutional environment. The *amount* of policy processes and outcomes related to teachers, the degree of coordination and integration *between* them, as well as the capacity to monitor and address issues and reforms in member states have increased markedly. The mechanisms, policies and instruments have evolved over the period, with processes related to the Semester and the ET 2020 Working Groups, for instance, having become more structured and formalised (A1, A5, A11). Shaping the scope and boundaries for issue framing and interaction, the elaboration of institutional infrastructure is critical to the bridging issue field of EU’s teacher governance, reflecting the dual aspiration to address horizontal issues as well as specific issues in member states. While not exhaustive of all developments, Figures 2 and 3 provide an overview of policies, mechanisms and instruments (see Table 2 for the distinctions in the coding agenda) with implications for teachers in the EU since 2007.

#### Policy mixes

Figure 2 indicates the striking development since the first EU schools policy was launched in 2007–2008 (EC, 2007a, 2008a). Mirroring the evolving issue framing, subsequent policies addressing teachers represent a continuation of the educational logic (EC, 2017a), yet a range of policies have served to incorporate issues such as digitisation (EC, 2013, 2018a, 2020a), socio-economic governance (EC, 2012a), skills and employment (EC, 2016a) and creating a European Education Area (EC, 2017c, 2020b). Moreover, the calls for reinforcing European identity and values (EC, 2017c, 2018b), following up on the Paris Declaration (EU Education Ministers, 2015), rekindle the essentialist notions of European culture informing EU discourses during the 1990s (Lawn and Grek, 2012; Walkenhorst, 2008). Characteristically, teachers constitute a cross-cutting theme in this evolving cluster of policies (A11), encapsulated by the fact that teachers and trainers constitute one of six central dimensions in the European Education Area, which brings together issues of lifelong learning, schools and higher education and skills, the UN Sustainable Development Goals (SDGs), the green and digital transitions, and the EU’s geopolitical positioning, within the context of post-pandemic recovery (EC, 2020b).

#### Governance mechanisms

Our analysis indicates a similar elaboration concerning governance mechanisms. Formally introduced in 2000, the Open Method of Coordination (OMC) remains in place as a distinctive mechanism of soft governance that seeks to align member state policies within a commonly agreed framework in policy areas subject to member state competences, such as education and employment (Dale, 2009). However, there have been major developments during the 2010s concerning cross-sectoral mechanisms with implications for EU teacher governance. While the most prominent example is the Semester (see below), other mechanisms include the ESSDE, the European Pillar of Social Rights and the Structural Reform Support Services (SRSS). The SRSS was launched in 2017 as part of the Commission’s Secretariat-General and upgraded in 2020 to the new separate DG Structural Reform Support (REFORM). The SRSS is demand-driven in the way that member states request tailored advice, which in some cases concern teachers. Drawing on its wide network as well as the Semester Country Teams, the SRSS indicates the Commission’s strengthened capacities in mobilising a diverse range of actors to support reform in member states (A12).

The Semester has brought together an array of policy domains in a single framework of socio-economic governance and mobilised actors at member state and European levels (A9, A10, B1). Teacher policy issues have figured in Semester Country Reports and National Reform Programmes for numerous member states during the 2010s, and CSRs related to, for instance, teacher education, professional development and attractiveness of the profession have been issued for a number of them, such as Czech Republic, Hungary, Malta and Slovakia. Characteristically, teacher and education policy issues are included as part of labour market issues in Country Reports and CSRs (A2, A5, A7, A8, A9, B1, B2; see also Stevenson et al., 2017).

The Semester has proved malleable in the way that it has been adapted to new priorities and policies, incorporating and serving as a lever for the coordination of other mechanisms and instruments (A4, A5, A9, A10, A14), such as the European Pillar of Social Rights, UN SDGs, and the Recovery and Resilience Facility (A7, A9). Moreover, the Semester’s identification of issues informs the SRSS’ evaluation of project requests (A12). The Semester has also come to include a strong investment angle, where the Commission highlights possibilities for using the European Social Fund and other instruments to address key issues, some of them concerning teachers (A2, A4, A5, A6, A7, A9, A14; EC, 2020b; see also European Court of Auditors, 2020).

At the same time, the EU Treaty’s distribution of competences continues to shape the institutional infrastructure, indicated by the boundaries for creating synergies between different mechanisms and instruments. For instance, linking the formally separate mechanisms of the Semester and the SRSS too closely would raise questions from member states (A12). Moreover, the Commission’s suggestion around 2015 to form an ET 2020 Working Group focusing on CSRs was never realised, since the interest among member states proved insufficient, given that CSRs tend to relate to specific conditions and the varying number of member states receiving education-related CSRs any given year (A11).

#### Policy instruments

The range of instruments addressing teachers’ learning, work and careers widened during the 2010s (see Figure 3). These different instruments together have the capacity to produce ‘interpretative’ as well as ‘resource’ effects, that is, they concern the exchange of knowledge, yet also the granting and shifting of incentives and resources (either material or symbolic) that might change administrative and policy processes (Marques, 2021).

The more education-focused instruments remain in place, yet they have developed and been complemented with new ones. The multiple generations of Working Groups, created by DG EAC from 2001 onwards and composed by experts appointed by EU member states and representatives of interest organisations, have always included a group focused on teachers (Stéger, 2014; Symeonidis, 2021). During the 2010s, the ET 2020 Working Groups were complemented with peer learning instruments, involving different formats of Peer Review and Peer Counselling, bringing together peers on the request of a member state to provide advice free of charge on specific policy developments (A11). The Lifelong Learning Programme was expanded and relabelled the Erasmus+ Programme in 2014, still supporting teachers’ professional development, mobility, and school and teacher education collaboration. Innovations introduced during the 2010s include the Education and Training Monitor, the annual European Education Summits, the Innovative Teaching Award and the digital platforms European Toolkit for Schools, School Education Gateway, and the SELFIE tool (Self-reflection on Effective Learning by Fostering the use of Innovative Educational technologies)(A11).

Furthermore, the level of instruments interaction (Marques, 2021) illustrates the evolution of EU teacher governance as a bridging issue field. For instance, the Education and Training Monitor, formally part of the ET 2020 framework, constitutes an important resource in the socio-economic governance mechanism of the Semester (A7, A8). The variety of EU funding instruments provide further examples of how EU’s governance of teachers straddles several policy domains. First, the ‘social prerogatives’ budget lines, managed by DG EMPL, support the dialogue, capacity building, and projects of European social partners, such as EFEE and ETUCE (B1, B2, C1). Second, the European Social Fund, also managed by DG EMPL and aiming to support job growth, continues to be extensively used to support teacher related activities (A8; see also Stéger, 2014; Symeonidis, 2021). Third, the groundbreaking European Recovery Instrument (Next Generation EU), in line with the Multi-annual Financial Framework 2021–2027, channels funds into education and training through the Erasmus+ Programme, Horizon Europe, Digital Europe, the InvestEU programme, the Recovery and Resilience Facility, the Technical Support Instrument, the European Structural and Investment Funds (including the European Social Fund Plus) and the European Regional and Development Fund (EC, 2020b; A8, A13).

## Discussion

This section discusses the findings regarding the scope, pace and linearity of change in EU governance of teachers between the mid-2000s and 2020, including how they correspond with Zietsma et al.’s (2017) hypothesis concerning change in bridging issue fields. They observe that the scope of change in such fields are likely to primarily produce convergent change that might add up to significant transformations over time. The pace of change is likely to be slow and non-linear, given that multiple different logics and actors with links to a variety of fields are assumed to form part of a bridging issue field. However, once actors agree on a given issue, changes are likely to diffuse rapidly in the bridging issue field and to related fields of professional exchange.

In discussing these questions, we need to consider the nature of the empirical material, which emphasises EU level developments as represented by the Commission. This material enables us to address the research questions, yet the complex multi-level nature of EU governance means that there are aspects of field change that we are not able to consider for this paper.

Our findings support the hypothesis of Zietsma et al. (2017) to a large extent, yet they also raise questions about how to make sense of convergence, changes and diffusion in the EU teacher policy field. The context of EU governance and the OMC provides specific conditions for the evolution of EU teacher governance as a bridging issue field, which is premised on bringing together different logics and actors within an institutional infrastructure that is designed, and obliged via the EU Treaty, to allow for contestation and diverse policies and practices in member states. In this sense, the evolution of EU teacher governance is defined by the intentional limits to the possibilities for imposing EU level convergence in policy and practice.

At the same time, the findings indicate changes over the period in terms of the elaboration of issue framings and institutional infrastructures as well as the mobilisation of actors and networks. The overall trajectory is characterised by incremental change although the launch of major policy initiatives and shocks, such as the financial crisis of 2008, are clearly apparent in the material. Over time, and in retrospect, the accumulation of incremental changes come to represent substantial evolution; the field of EU teacher governance anno 2020 is very different from the one in 2007. Considering the concept of field structuration (DiMaggio and Powell, 1983; Martin, 2003), it would be relevant to further analyse socialisation and alignment of organisations in the EU teacher policy field. Due to the different logics and types of actors co-constituting the field, as well as the nature of the empirical material, we do not want to overstate the extent to which the field structuration in EU teacher governance has led to convergence in terms of shared identities. On the one hand, formal units of governance, field coordinators, institutional entrepreneurs and arbiters of taste, with different horizons of action, have been mobilised, suggesting an intensified pursuit of interests via the elaborated institutional infrastructure and thus a widening awareness regarding EU governance as a ‘common enterprise’. Yet, on the other hand, the findings correspond with the theoretical assumption that contestation and multiple logics are integral to bridging issue fields, resulting in slow and non-linear change processes. Due to the many different interests at stake, the issue framings, and associated logics of education, employment, economy and governance, remain contested, although the elaboration of institutional infrastructure suggests an evolution towards a more comprehensive governance logic and hence ‘rules of the game’ in the field.

We understand the field changes as policy-led in the sense that the institutional infrastructures and issue framing have determined the scope for field agency and access. Zietsma et al. (2017) observe that a less elaborated institutional infrastructure entails weaker field boundaries, enabling entry of new actors and thereby providing more space for innovation. Yet, the institutional infrastructure put in place in the EU teacher policy field is explicitly meant to promote and diffuse innovation in the field, but we might add that this is a more steered or controlled form of innovation.

Our findings correspond with the theoretical observation that professional exchange field actors are often drawn into issue fields because of regulatory shifts, new policies and the creation and legitimisation of new actors. While the trajectory of EU teacher governance goes back at least to the 1980s (Poissonneau and Nolda, 2012; Sayer, 2006; Symeonidis, 2021), our study corroborates existing evidence regarding the importance of the Lisbon Strategy (Lawn and Grek, 2012; Pépin, 2011) in amplifying education and training policies and shaping issue framing and institutional infrastructure throughout the 2000s, including for teachers.

In this respect, our finding that the Commission has been the main boundary organisation in the field should be understood in the context of the mid-term review of the Lisbon Strategy which strengthened the political role of the Commission (Borrás, 2009; Robertson, 2008). By highlighting its capacity-building and field agency over the period, our analysis adds pertinent insights to the roles and modus operandi of the Commission, as a single unit comprising numerous DGs, in contemporary EU governance. However, it would be misleading to represent the Commission as monolithic, as the documents and interviews indicate that the DGs are not fully aligned due to lack of communication and mutual positioning. Such non-alignment is, for instance, reflected in the different emphases in the Communication (EC, 2012a) and associated Staff Working Document (EC, 2012b), the latter of which, in contrast to the former’s focus on skills and employability, emphasised the competences needed for life and developing human potential fully.

Several of our findings demonstrate the theoretical point that actual results of policy-led changes depend on the interests and ability of actors to resist implementation (Zietsma et al., 2017). In the bridging issue field of EU teacher governance, we understand the relatively slow and non-linear field changes as reflecting actors’ different interests and levels of engagement with EU policies and policymaking, in turn pointing towards the continuing challenge of mobilising actors and diffusing policy outcomes in the context of EU soft governance.

## Concluding remarks

Transnational governance fields remain little studied using neo-institutional field theory. In demonstrating the ways in which EU teacher governance forms a bridging issue field, this paper contributes to the scholarly debate on EU governance. In theoretical terms, we argue that neo-institutional field theory enables epistemic gains related to the explanation of the characteristics underpinning the bridging issue field of EU teacher governance, including the field’s slow, non-linear evolution in a specific context of soft governance, as different types of actors are mobilised and respond to policy issues, with the Commission in the role as the main boundary organisation reaching deep into teachers’ professional exchange fields in member states.

This theoretical point raises two sets of research issues. First, while the empirical material underpinning our analysis does not allow for considering the evolving relations between EU teacher governance and specific member states in depth, the interviews suggest varying levels of engagement among member state governments over the period, for instance between the UK and some Central and Eastern European member states (A5, A7, A8). In this respect, it is also remarkable that the study suggests that member states’ Ministers of Education only more recently have become more engaged in EU governance, trailing behind Ministers of Finance and State who dominated the representation of member states in debates about education and teachers in the austerity context of the early 2010s. Moreover, there are large differences between member states regarding the use of the European Social Fund to support teaching-related activities (A8). These issues merit more detailed investigation as does the actual impact of the Semester in member states (Sorensen et al., 2021). The different forms of contestation and adaptation of EU policy recommendations in national policies and practice thus constitute a distinctive research agenda, all the more pertinent given the recent Recovery Plans, underpinned by member states’ common loans, which have had the effect of making the distribution of incentives conditional on the implementation of EU recommendations. Likewise, while the institutional infrastructure, especially via the Semester and the ESSDE, has come to provide openings and incentives for teacher unions, employers, and other interest organisations to seek influence on EU policies, the level of their engagement varies strongly in practice (Sorensen et al., 2021). In theoretical terms, this set of research issues concerns the degree to which the EU’s institutional infrastructure is invested with authority by different types of actors, thereby enabling such infrastructure to actually work as integrative forces in the field (cf. Jochim and May, 2010). In this respect, comparative studies between the EU and the OECD, which has less political leverage and therefore arguably is subject to less scrutiny and accountability than the Commission (A2), would also help to shed further light on the European and global governance of teachers.

The second set of research issues concerns the relations between transnational bridging issue fields and professional exchange fields. Making sense of the contingent relationships between these types of fields, and the conditions that make them possible in terms of framing, actors and institutional infrastructures, merit further research. In light of the EU’s continuous efforts from the 1980s onwards to promote teacher’s learning and labour market mobility in Europe (cf. Directive 2013/55/EU on the recognition of teacher qualifications for free movement in the Single Market), we might even understand the trajectory of EU governance over recent decades as strategic efforts to create a European professional exchange field for teachers, given further momentum by the strategy to create a European Education Area (Alexiadou and Rambla, 2023). This field remains rudimentary, yet the point highlights the need to trace the evolution of issues, such as teacher mobility, shortages, recruitment, and retention, as this European professional exchange field develops and adds further complexity to the very issues that EU governance seeks to address.

## Appendix

### Empirical material

#### Policy documents: European Commission (EC) communications and staff working documents

1. EC (2007a). *Commission Staff Working Document: Schools for the 21st Century.* SEC(2007) 1009. Brussels, 11.07.2007.
2. EC (2007b). *Improving the Quality of Teacher Education.* COM(2007) 392 final. Brussels, 3.8.2007.
3. EC (2008a). *Improving competences for the 21st Century: An Agenda for European Cooperation on Schools.* COM(2008) 425 final. Brussels, 3.7.2008.
4. EC (2008b). *Commission Staff Working Document accompanying the Communication from the Commission ‘Improving competences for the 21st Century: An Agenda for European Cooperation on Schools’.* SEC(2008 )2177. Brussels, 3.7.2008.
5. EC (2012a). *Rethinking Education: Investing in skills for better socio-economic outcomes.* COM(2012) 0669 final. Strasbourg, 20.11.2012.
6. EC (2012b). *Commission Staff Working Document 'Supporting the Teaching Professions for Better Learning Outcomes' Accompanying the document Communication from the Commission Rethinking Education: Investing in skills for better socio-economic outcomes.* SWD(2012) 374 final. Strasbourg, 20.11.2012.
7. EC (2013). *Communication from the Commission to the European Parliament, the Council, the European Economic and Social Committee and the Committee of the Regions. Opening up Education: Innovative teaching and learning for all through new Technologies and Open Educational Resources.* COM(2013) 654 final. Brussels, 25.9.2013.
8. EC (2016a). *Communication from the Commission to the European Parliament, the Council, the European Economic and Social Committee and the Committee of the Regions – A new skills agenda for Europe: working together to strengthen human capital, employability and competitiveness.* Brussels, 10.6.2016.
9. EC (2016b). *Communication from the Commission to the European Parliament, the Council, the European Economic and Social Committee and the Committee of the Regions – Improving and modernising education.* Brussels, 7.12.2016.
10. EC (2017a). *School development and excellent teaching for a great start in life*. COM(2017) 248 final. Brussels, 30.5.2017.
11. EC (2017b). *Commission Staff Working Document Accompanying the document Communication on school development and excellent teaching for a great start in life.* SWD(2017) 165 final. Brussels, 30.5.2017.
12. EC (2017c). *Strengthening European Identity through Education and Culture*. The European Commission’s contribution to the Leaders’ meeting in Gothenburg, 17 November 2017. COM(2017) 673 final. Strasbourg, 14.11.2017.
13. EC (2018a). *Communication from the Commission to the European Parliament, the Council, the European Economic and Social Committee and the Committee of the Regions on the Digital Education Action Plan.* COM(2018) 22 final. Brussels, 17.1.2018.
14. EC (2018b). *Proposal for a Council Recommendation on promoting common values, inclusive education, and the European dimension of teaching.* COM(2018) 23 final. Brussels, 17.1.2018.
15. EC (2020a). *Communication from the Commission to the European Parliament, the Council, the European Economic and Social Committee and the Committee of the Regions Digital Education Action Plan 20212027: Resetting education and training for the digital age.* COM(2020)624 final. Brussels, 30.9.2020.
16. EC (2020b). *Communication from the Commission to the European Parliament, the Council, the European Economic and Social Committee and the Committee of the Regions on achieving the European Education Area by 2025.* COM(2020)625 final. Brussels, 30.9.2020.

#### Interview data

Nineteen semi-structured research interviews, based on interview guides tailored to each interview, and conducted in the period from February 2019 to July 2021.

- All 19 interviews were individual, except interview A8, A14, D1 and E1 (see overview below)
- The interviews were conducted face to face, except D1 (written communication), A13, A14, B3 and E1 (online meeting).
- All interviews were recorded and transcribed, except A10, A12, A14 and D1. These were not recorded due to restrictions. Instead, notes were taken and shared with interview participants who approved them.

In addition, *three exploratory background interviews* were conducted with the European Commission Representation in Belgium about the European Semester and consultation with stakeholders; a policy officer in DG EMPL concerning the assessment of Country-Specific Recommendations of the European Semester; and a senior executive from a major consultancy with experience in undertaking projects for the Commission.

**Table:**

Interview participant’s role in organisation	Interview code
*European Commission, DG Education and Culture*	
Former Policy Officer Schools and Teachers	A1
Senior Manager	A2
Policy Officer Higher Education	A3
Policy Analyst Schools	A5
Country Desk Officer I	A7
Country Desk Officer II	A8
Country Desk Officer III and Coordinator of European Structural Investment Funds	A8
Country Desk Officer IV	A9 and A13
Policy Officer Schools and Teachers	A11
*European Commission, DG Employment, Social Affairs and Inclusion*	
Coordination Policy Officer	A4
European Semester Policy Officer	A6
*European Commission, Secretariat-General*	
Coordination Policy Officer	A10
Team Leader, Structural Reform Support Service	A12
Senior Expert	A14
*European Commission, DG Communication*	
Policy Officer	A14
*European Trade Union Committee for Education (ETUCE)*	
ETUCE policy officer	B1
Senior representative	B2
Former senior representative	B3
*European Federation of Education Employers (EFEE)*	
Senior representative	C1
*European Parliament*	
Research Administration, Policy Department B: Structural and Cohesion Policy	D1
*European network for teacher educators*	
Network board member	E1

## Notes

1. For policy documents, where the European Commission is abbreviated to EC, these are listed in the Appendix.

2. These codes refer to interviews (see Appendix).

3. For clarification, the European Council and Council of the EU serve different purposes. Convening the Heads of government from EU member states, the European Council defines the EU’s political direction. Meanwhile, the Council of the EU comprises government ministers, grouped by policy area, such as education or employment. These Councils negotiate EU legislation and regulations (European Council, 2023).
